# Rhabdomyolysis in Critically Ill Patients With COVID-19: A Retrospective Study

**DOI:** 10.7759/cureus.37333

**Published:** 2023-04-09

**Authors:** Abdulqadir J Nashwan, Anood Alassaf, Ahmad A Abujaber, Mohammad Al Wraidat, Dore C Ananthegowda, Salma K Al-Kaabi, Muftah Othman, Muayad K Ahmad, Muna Al Maslamani, Mohamad Khatib

**Affiliations:** 1 Nursing Department, Hamad Medical Corporation, Doha, QAT; 2 Pediatrics Department, Hamad Medical Corporation, Doha, QAT; 3 Internal Medicine Department, Hamad Medical Corporation, Doha, QAT; 4 Critical Care Department, Hamad Medical Corporation, Doha, QAT; 5 Preventive Medicine, Ministry of Public Health, Doha, QAT; 6 Nephrology, Hamad Medical Corporation, Doha, QAT; 7 Medicine, Hamad Medical Corporation, Doha, QAT; 8 Infectious Diseases, Hamad Medical Corporation, Doha, QAT; 9 Critical Care Medicine, Hamad Medical Corporation, Doha, QAT

**Keywords:** renal replacement therapy, critical care, sars-cov-2, covid-19, rhabdomyolysis

## Abstract

Introduction: The total number of ICU admissions for COVID-19 patients has increased steadily. Based on the research team’s clinical observations, many patients developed rhabdomyolysis, but few cases were reported in the literature. This study explores the incidence of rhabdomyolysis and its outcomes, like mortality, the need for intubation, acute kidney injury, and the need for renal replacement therapy (RRT).

Methods: We retrospectively reviewed the characteristics and outcomes of patients admitted to the ICU at a COVID-19-designated hospital in Qatar between March and July 2020. Logistic regression analysis was used to determine factors associated with mortality.

Results: 1079 patients with COVID-19 were admitted to the ICU, and 146 developed rhabdomyolysis. Overall, 30.1% died (n = 44), and 40.4% developed Acute Kidney Injury (AKI) (n = 59), with only 19 cases (13%) recovering from the AKI. AKI was significantly associated with increased mortality rates among rhabdomyolysis patients. Moreover, significant differences were found between groups regarding the subject's age, calcium level, phosphorus level, and urine output. However, the AKI was the best predictor of mortality for those who got the COVID-19 infection and rhabdomyolysis.

Conclusion: Rhabdomyolysis increases the risk of death in COVID-19 patients admitted to the ICU. The strongest predictor of a fatal outcome was acute kidney injury. The findings of this study emphasize the importance of early identification and prompt treatment of rhabdomyolysis in patients with severe COVID-19.

## Introduction

In December 2019, severe acute respiratory syndrome coronavirus-2 (SARS-CoV-2) was identified as the new coronavirus that causes coronavirus disease (COVID-19) [1]. Patients with viral pneumonia had symptoms similar to SARS-CoV-2 and Middle East respiratory syndrome coronavirus (MERS-CoV) infections, such as fever, breathing difficulties, and bilateral lung infiltration in the most severe forms [2-5]. Several researchers have developed a management algorithm based on previous experience treating SARS and MERS virus-induced severe respiratory failure [3]. Initial reports from China, Italy, and the United States indicated a high death rate and a lack of intensive care unit (ICU) capacity [6-7]. Every year, the critical care community gains expertise in managing severe acute respiratory infections caused by unknown sources. The foundation for managing critically ill COVID-19 patients should be rooted in this evidence-based approach while guaranteeing the insights gained from every patient are maximized to support the treatment of those who come after them [8-10]. Rhabdomyolysis (RML) is a syndrome that occurs when skeletal muscle cells disrupt and release creatine phosphokinase (CPK), lactate dehydrogenase (LDH), and myoglobin into the interstitial space and plasma [11]. Myalgia, muscular weakness, and myoglobinuria are common clinical presentations of RML. The causes of RML could be genetic or acquired [12]. Infections are not a common cause of RML; however, Influenza A and B viruses are known causes of RML [13], and only occasional case reports about RML in COVID-19 have been reported [14-16]. This is a retrospective, descriptive study of the incidence of RML and its outcomes, like mortality, need for ventilation and tracheostomy, acute kidney injury, and need for renal replacement therapy (RRT) in patients with COVID-19.

## Materials and methods

Ethical considerations

The Medical Research Center of Hamad Medical Corporation approved this project (MRC-01-20-857). The research was carried out in compliance with the ethical principles outlined in the Helsinki Declaration of 1964 and its subsequent modifications and related ethical norms. Due to the study's retrospective nature, no consent was obtained.

Data collection

The retrospective data collection for this study was conducted over five months, from March to July 2020. The data were collected from patients admitted to the critical care unit of Hazm Mebaireek General Hospital (HMGH), a member of Hamad Medical Corporation. HMGH was designated as the main COVID-19-designated facility during the pandemic in Qatar. Therefore, all cases of respiratory distress and multi-organ failure due to COVID-19 were admitted to this hospital.

The data collection process involved reviewing the medical records of all patients who were admitted to the critical care unit during the specified period. The medical records were reviewed by trained research assistants, who were responsible for collecting the necessary data. The data collected included information such as age, sex, comorbidities, presenting symptoms, laboratory investigations, radiological findings, and the clinical course of the disease.

In addition to the data collected on COVID-19 patients, baseline demographic information was also gathered. This included demographic characteristics such as age, sex, occupation, and underlying health conditions that may have predisposed individuals to more severe COVID-19 disease.

Eligibility criteria

The inclusion criteria for this study focused on critically ill COVID-19 patients with laboratory evidence of rhabdomyolysis (RML). Patients who were 18 years or older and had a confirmed laboratory diagnosis of COVID-19 were included in the study. Only patients admitted to the critical care units of Hazm Mebaireek General Hospital (HMGH) for more than 24 hours were included. Patients with CPK levels greater than five times the normal limit (1500-100,000 IU/L) and myoglobin levels greater than 80 ng/mL were included based on laboratory evidence of RML [17]. Concurrent conditions such as pregnancy were excluded to ensure that the study only included patients unaffected by confounding factors that could have influenced the development of rhabdomyolysis in COVID-19 patients. These inclusion criteria were carefully chosen to ensure the study results were relevant and accurate for the target patient population.

Study outcomes

The primary outcome of this study was to determine the incidence of rhabdomyolysis (RML) in critically ill COVID-19 patients and its association with mortality. The study aimed to investigate the prevalence of RML in this patient population and the impact of this condition on patient outcomes. The primary outcome was assessed by analyzing the laboratory data of patients admitted to the critical care units of Hazm Mebaireek General Hospital (HMGH) during the study period. Mortality was evaluated as a secondary outcome and was compared between patients with and without RML.

Statistical analysis

A univariate analysis was conducted to determine the variables significantly associated with the outcome variable, i.e., mortality. The Chi-square test was used to compare qualitative data, whereas the independent t-test was used to evaluate quantitative data. The eligible variables were then incorporated into the step-wise logistic regression model. A p-value greater than 0.1 was utilized to exclude variables from the first multiple-regression model. Statistical significance was defined as two-sided p-values less than 0.05. The correlation matrix and variance inflation components were used to test for multicollinearity. IBM Corp. Released 2021. IBM SPSS Statistics for Windows, Version 28.0. Armonk, NY: IBM Corp was used for all analyses.

## Results

Between March and July 2020, 1079 COVID-19 patients were admitted to the ICU. One hundred and forty-six of them developed RML. All cases were reviewed retrospectively, and the needed data was obtained utilizing electronic health records (EHRs).

Sample characteristics

The average age in the mean (SD) was 51 (±15.0) years old. Most of the study sample were males 95.9% (n=140) (this might be related to the typical demographics of the Gulf region, where most of the patients are males, as well as our hospital, is located near an industrial area where the majority of clients are male workers), and only 4.1% were females (n=6). Among the entire study sample (n=146), 30.1% died (n= 44), and 69.9% survived (n=102). In addition, 41.1% of the study sample was diagnosed with DM (n=60), the same percentage with HTN (n=60), and 40.4% with AKI (n=59). At the same time, only 19 of them recovered from the AKI; 85.6% were intubated (n=125), and 8.2% had chronic kidney disease (CKD) (n=12) (Table 1).

**Table 1:** Demographic characteristics of the sample DM: Diabetes Mellitus; HTN: Hypertension; CKD: Chronic Kidney Disease; AKI: Acute Kidney Injury; SLED: Sustained Low-Efficiency Dialysis; CRRT: Continuous Renal Replacement Therapy.

Variables	Options	N	%
Gender	Male	140	95.9%
	Female	6	4.1%
DM	Yes	60	41.1%
	No	86	58.9%
HTN	Yes	60	41.1%
	No	86	58.9%
CKD	Yes	12	8.2 %
	No	134	91.8%
AKI	Yes	59	40.4 %
	No	87	59.6%
Intubation	Yes	125	85.6%
	No	21	14.4%
Recovery from AKI	Recovered	19	13.0%
	Not recovered	40	27.4%
Dialysis modality	Not dialyzed	9	6.2%
	SLED	28	19.2%
	CRRT	3	2.1%
	SLED/CRRT	19	13%
Mortality	Alive	102	69.9%
	Died	44	30.1%

The laboratory investigations for the RML patients are shown in Table 2.

**Table 2:** The laboratory investigations for the rhabdomyolysis patient HLH: Hemophagocytic lymphohistiocytosis; LDH: Lactate Dehydrogenase; CPK: Creatine phosphokinase; ALT: Alanine transaminase; AST: Aspartate aminotransferase; SD: Std. deviation

	Age	HLH Score	LDH	CPK	Creatinine	Potassium	Calcium	Phosphorus	Myoglobin	Urine output	ALT	AST
Valid	146	145	146	146	146	146	146	146	146	140	146	146
Mean	51.26	52.52	711.82	2663.87	170.99	4.36	2.24	1.29	1398	1559.36	164.13	289.26
Median	50.00	63.00	615.50	1698.00	97.500	4.30	2.28	1.10	820	1485.00	62.50	90.50
SD	14.99	31.87	374.26	3308.13	277.31	.820	.262	.739	1900	843.288	400.11	906.49

By testing the association between the mortality rate among RML patients and AKI, the Chi-Square test revealed a significant association between the AKI and mortality rate p< 0.001; as 40% of the RML patients developed AKI (n=59), 67.8% of them died (n=40), and 32.2% of them survived (n=19). On the other hand, for those who had RML but not AKI (n=87), only 4.6 % of them died (n=4). However, the association between the mortality rate among RML patients and DM, HTN, and CKD revealed no significant association with a mortality rate p> 0.05, p< 0.071, and p> 0.05, respectively.

Step-wise logistic regression analysis was used to control the effect of confounding variables. The overall goodness-of-fit was satisfactory. The regression model significantly predicts that AKI is the best predictor of mortality rate, with a 95% Cl of 5.640 to 58.409; the likelihood that a patient will die provided that he/she sustained AKI is more than 18 times higher than those who didn’t sustain AKI, p<0.001 (Table 3).

**Table 3:** Logistic regression analysis a. Variable(s) entered on step 1: Age, CPK, calcium, phosphorus, urine output, AKI. CPK: Creatine phosphokinase; AKI: Acute kidney injury. OR: Odds ratio

								95% CI for EXP(B)	
		B	S.E.	Wald	df	Sig.	OR	Lower	Upper
Step 1^a^	Age	.032	.018	3.310	1	.069	1.033	.997	1.070
	CPK	.000	.000	.012	1	.911	1.000	1.000	1.000
	Calcium	.339	1.196	.081	1	.777	1.404	.135	14.623
	Phosphorus	-.003	.351	.000	1	.992	.997	.501	1.981
	Urine output	-.001	.000	4.388	1	.036	.999	.999	1.000
	AKI	2.899	.596	23.630	1	.000	18.151	5.640	58.409
	Constant	-3.921	2.918	1.806	1	.179	.020		

## Discussion

Rhabdomyolysis (RML) is the clinical and chemical manifestation of myocyte necrosis, breakdown, and the release of intracellular content into circulation. It can happen for multiple reasons; however, in our paper, COVID-19 infection in patients admitted to the critical care unit is the reason of interest. Manifestations, like urine color change, can be subtle or serious, leading to renal failure and arrhythmias [18-20]. Those manifestations are reflections of the acute release of multiple electrolytes and enzymes, which at substantially raised levels, affect multiple body systems. For example, a high level of free myoglobin released from the myocyte can precipitate in the glomerular membrane of the kidney, impair filtration, and cause tubular necrosis, leading to kidney injury [21]. Another feared complication is hyperkalemia, which, at high levels, can lead to fatal arrhythmias. Other cellular components that leak into the circulation are aldolase, phosphate, myoglobin, CK, lactate dehydrogenase, aspartate transaminase, and urate, of which CK is the most sensitive marker for the degree of injury [21,22].

COVID-19 has often been reported to be associated with RML in patients under active treatment with hydroxychloroquine [16] and oseltamivir [21] and at the initial presentation with no previous therapy [23]. Khan and colleagues have provided in their literature review an insight into the proposed mechanisms behind infection-induced RML. In cases of viral infection, these include hypoxemia (caused by sepsis, hypoxia, dehydration, acidosis, electrolyte disturbances, and hypophosphatemia), dysfunction in cellular energy utilization, glycolytic enzyme activity, and activation of lysosomal enzymes [22]. Others attribute musculoskeletal injury in COVID-19 infection to indirect host cytokine storm immune response [1]. The result will be the disruption of ATP-dependent ion channels, which eventually leads to an influx of calcium into the cell, activating proteolytic enzymes and destroying cell membrane proteins [24,25].

Few research studies were published related to RML in COVID-19 infection; most were case reports and case series. In some cases, RML was the initial presentation of COVID-19 infection; in others, it was a late manifestation [26]. Our single-center retrospective study examined the patients in the critical care unit in Qatar and found that the incidence of RML in COVID-19 patients is 13.5%, with a mortality rate of 30.1%. While the incidence of RML seems lower than the one generally reported in Qatar (15.7%), the mortality rate is almost double what was reported by Ali et al. (13.8%) [1]. Comparing these results with those in other countries, Haroun et al. conducted a single-center study in the Bronx, New York City. They found that the incidence of RML among all COVID-19 patients was 16.9%, with an in-hospital mortality rate of 47.1% [2]. When comparing different studies on the incidence and mortality rates of COVID-19, several factors may contribute to differences in the results. These include population characteristics, healthcare infrastructure, study design and sample size, timing, and disease severity. For example, a study in Qatar found a lower incidence of RML but a higher mortality rate than another study conducted in the Bronx, New York City. To better understand the discrepancies in incidence and mortality rates, it is essential to thoroughly compare these studies, taking into account these factors and any other relevant variables. This will help inform future research and improve patient care and management strategies.

In our study, AKI emerged as a significant complication in patients with COVID-19 who developed RML, with an incidence of around 40%. The results revealed that AKI was significantly associated with higher mortality in those patients. The analysis showed that AKI was the best predictor of a fatal outcome (95% Cl of 5.640 to 58.4). This adds another reason to explain the increased mortality rate among COVID-19 patients, especially those whose illness is complicated by RML. Our study agrees with Geng et al., who considered AKI an important prognostic factor in cases where creatinine kinases were rising, indicating patients’ deterioration and muscle necrosis [7]. Other comorbidities (DM, HTN, and CKD) did not significantly influence the mortality rate.

Among the many markers of muscle injury, creatinine kinase levels can be significantly elevated, even without clear clinical signs of RML. Creatinine kinase also correlates with the severity of muscle injuries and patients’ illnesses. It has a serum half-life of 36 hours. It drops at a relatively steady rate of 40 to 50 percent every day once the reason for the muscle injury has been eliminated and the patient is being managed properly [27]. In our study, the mean CK level was 2663 units\L (normal range: 55-170 units\L in males, 30-135 units\L in females), which is higher than reported in other studies [7].

The study has multiple limitations, mainly related to the nature of the retrospective design. The probability of missing data might affect the findings. The study sample was obtained from a single center, which may not give a representative sample of the population of Qatar. The majority of cases were male, which might be related to the typical demographics of the Gulf region, where most of the patients are men. Our hospital is located near an industrial area where most clients are male workers.

## Conclusions

The study findings suggest that RML is associated with a higher mortality rate among COVID-19 patients admitted to the ICU. Acute kidney injury (AKI) is a strong predictor of a fatal prognosis. This highlights the critical need for early detection and prompt management of RML to improve the outcome of critically ill COVID-19 patients. Future research is warranted to explore further the mechanisms underlying RML-associated mortality and identify effective interventions that can improve patient outcomes in this population. These findings underscore the urgency of addressing RML as a crucial factor in managing severe COVID-19 cases.
